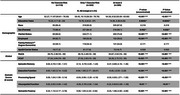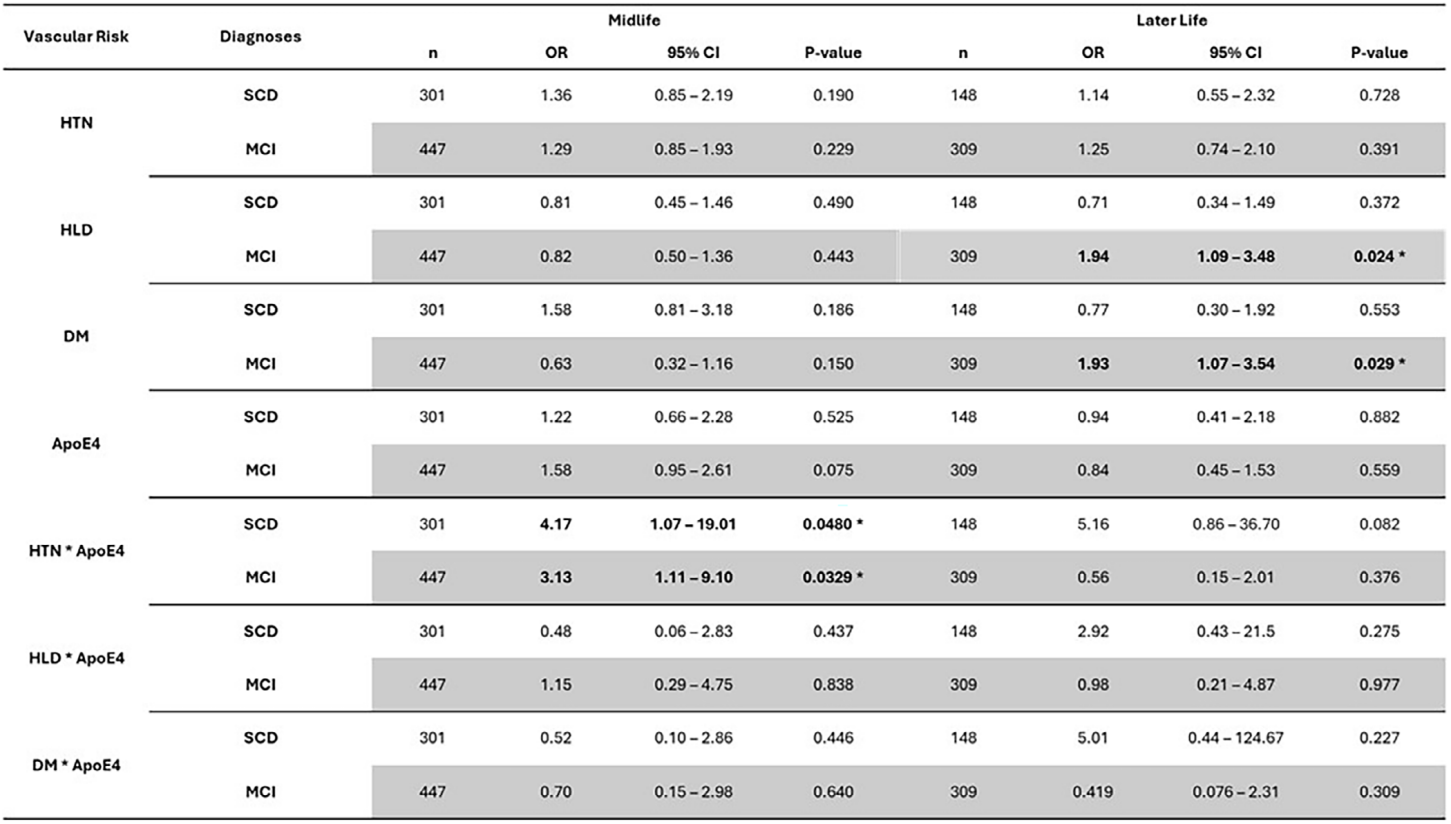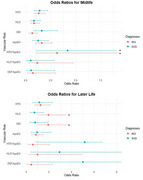# Impact of Vascular Risk Burden and ApoE4 on Cognitive Impairment: Insights from a Non‐Demented Southeast Asian Population

**DOI:** 10.1002/alz70856_102435

**Published:** 2025-12-26

**Authors:** Smriti Ghildiyal, Ashwati Vipin, Gurveen Kaur Sandhu, Pricilia Tanoto, Nagaendran Kandiah

**Affiliations:** ^1^ Dementia Research Centre (Singapore), Lee Kong Chian School of Medicine, Nanyang Technological University, Singapore, Singapore; ^2^ Lee Kong Chian School of Medicine, Nanyang Technological University, Singapore, Singapore; ^3^ Neuroscience and Mental Health Programme, Lee Kong Chian School of Medicine, Nanyang Technological University, Singapore, Singapore; ^4^ National Healthcare Group, Singapore, Singapore

## Abstract

**Background:**

Factors contributing to the progression of cognitive decline include age, lower education, vascular risk factors: hypertension(HTN), hyperlipidemia(HLD), diabetes mellitus(DM), and Apolipoprotein E4(ApoE4). The interaction between aging and vascular processes increases the risk of cognitive impairment and neurological disorders. Similarly, ApoE4 is a major genetic risk for late‐onset Alzheimer's Disease. The interaction between vascular risk factors and ApoE4 status on cognitive impairment requires further exploration.

**Method:**

838 non‐demented participants(Age=60.36±10.55) were recruited in the community‐based BIOCIS cohort. The cohort was stratified based on vascular risk: no vascular risk(*n* = 110), one vascular risk(*n* = 343), multiple vascular risks(*n* = 385). Subsequent analysis was stratified into midlife(45‐65, *n* = 448) and later life(>65, *n* = 310).

Participants were administered global cognitive tests (MoCA, VCAT), and comprehensive neuropsychological assessments including episodic memory(EM), executive function(EF), processing speed(PS), visuospatial function(VS) and semantic fluency(SF)(transformed into z‐scores). Participants were classified as cognitively normal(CN), subjective cognitive decline(SCD) and mild cognitive impairment(MCI) according to Petersen's‐(2004) and NIA‐AA criteria. Vascular risk factors included clinical history(HTN, HLD, DM), medication consumption, systolic blood pressure, total cholesterol and fasting HBA1C. ApoE allelic variation was determined via Taqman SNP genotyping qRT‐PCR methodology.

**Result:**

Participants with increasing vascular risk burden were older(*p* <0.001) and less educated(*p* = 0.024) and displayed poorer cognitive performance across cognitive domains: MoCA, VCAT, EM, EF, PS, SF(all *p* <0.001) and VS(*p* <0.01).

Individually, risk factors such as HLD and DM have a significant impact on cognitive impairment(CI). In later life, the odds of MCI classification are nearly doubled in individuals with HLD(OR=1.94, 95%CI=1.09–3.48, *p* = 0.024) and DM(OR=1.93, 95% CI=1.07–3.54, *p* = 0.029).

In ApoE4 carriers, HTN significantly increases the odds of CI. In midlife, the interaction between HTN and ApoE4 quadruples and triples the risk of SCD(OR=4.17, 95% CI=1.07–19.01, *p* = 0.048) and MCI(OR=3.13, 95% CI=1.11–9.10, *p* = 0.033) respectively. All results remain significant despite controlling for age, education and gender.

**Conclusion:**

In a non‐demented Southeast Asian cohort, higher vascular risk factor count heightens the risk of cognitive impairment. While individually HTN, unlike HLD and DM, does not directly impact cognitive syndrome classification, in conjunction with ApoE4, it substantially increases the likelihood of cognitive impairment in midlife. For ApoE4 carriers, aggressive treatment of HTN is imperative to prevent progression of cognitive impairment.